# Sustainable development Universities—Opportunities & challenges on the road to society 5.0 from the key stakeholder perspective

**DOI:** 10.1371/journal.pone.0308929

**Published:** 2024-08-15

**Authors:** Joanna ROSAK-SZYROCKA, Krzysztof KNOP

**Affiliations:** Faculty of Management, Czestochowa University of Technology, Czestochowa, Poland; University of Pardubice: Univerzita Pardubice, CZECHIA

## Abstract

There has been a growing push on universities worldwide to demonstrate how their work contributes to the indicators of sustainable development goals. In addition to producing a foundation of human resources to assist the change toward greater sustainability, universities may have a significant influence on individual behaviour. The article’s goal is to highlight the potential and difficulties that the surveyed universities face as they work to construct a 5.0 society and pursue sustainable development. It does this by analysing students’ perspectives from these universities in ten different nations. A Computer-Assisted Web Interviewing (CAWI) questionnaire was used for the study. The hypotheses about the relationship between the university’s legal status and form of ownership and the level of students’ awareness of sustainable development were verified. The findings indicated that by promoting sustainable development, the universities under investigation had the opportunity to garner interest and involve students. Nevertheless, this calls for funding, better educational initiatives, and a well-rounded strategy. Furthermore, encouraging a sustainable culture within the university ecosystem and openly sharing these efforts with students and the general public will make universities more visible, respected, and driven, boosting involvement and engagement in sustainability initiatives on campus.

## 1. Introduction

Universities must take the lead in global sustainable development projects because of their crucial role in shaping society policy and teaching the next generation [1]. Encompassed of social, environmental, and human development goals, the Sustainable Development Goals (SDGs) provide a comprehensive agenda [2]. The growing trend of higher education in the context of sustainable development may be ascribed to three distinctive features of universities that make them particularly appealing for environmental and sustainability teaching [3]. Firstly, universities play a critical role in generating the human capital required for the shift to sustainability [4]. They also train a large number of professionals who will steer significant social institutions and make important policy choices [1]. Secondly, the research-intensive nature of SD is the second aspect behind the move to higher education. Studies that have already been written on SD have often stressed the need of research in advancing sustainability initiatives (United Nations, 2012; United Nations, 2015). Following the Rio+20 Conference, a publication titled "The Future We Want" emphasized how vital innovation and research are to implementing the concepts of sustainable development. Berchin et al. [5] assert that research is a fundamental component of universities and facilitates the exchange of information, which in turn drives social transformations. Research on sustainable development facilitates the streamlining of procedures and the comprehension of best practices, so offering essential backing to the progress made in the direction of sustainable development [4]. The pursuit and publication of research facilitates institutional knowledge-sharing and innovation, both of which are necessary for the achievement of sustainable development objectives [5]. Moreover, institutions have the opportunity to educate sustainable development while doing research, adding to the expanding corpus of global knowledge on sustainability. The consequent benefit is that students may use what they have learned in class to reinforce their comprehension of important sustainability principles in research settings [6]. The third component of universities is their network of intimate relationships with other universities as well as with entities in the public and commercial sectors. These networks make it possible to work together effectively on significant sustainability projects.

The article’s goal is to highlight the potential and difficulties that the surveyed colleges face as they work to construct a 5.0 society and pursue sustainable development. It does this by analyzing the perspectives of students from these universities in ten different nations. There are many studies on SD and society 5.0, but they are focused on the perspective of SD by universities [7] and students (Adhikari and Shrestha, 2023; Lee et al., 2023; Raza et al., 2023) or from the perspective of the application of technology, including the needs of society 5.0 towards SD or industry 4.0 [8–10]. Our study is novel. Our manuscript fills the research gap and can be a guide for universities in adapting their educational offerings to the needs of students. The following hypotheses were verified:

- H_0_: there is no relationship between the legal status and form of ownership of the university and the level of students’ awareness of sustainable development;
- H_1_: there is a relationship between the legal status and form of ownership of the university and the level of students’ awareness of sustainable development.

The hypotheses were verified using the nonparametric Pearson Chi-square test for 2×2 tables.

## 2. Literature review

Sustainable development Universities (SDU) aim to change the approach to education to one that integrates principles, values, and practices of sustainable development and needs to be incorporated into all forms of learning and education [11, 12]. All kinds of learning and education must embrace the concepts, values, and practices of sustainable development (Sustainable Development University), which seeks to transform the way that education is delivered. In order to achieve environmental, economic, social, and cultural sustainability in their particular countries, SDU may help people modify their behaviour [13, 14]. The following five components make up SDU [15, 16]: 1. instruction that enables students to gain the abilities, information, values, and skills necessary to guarantee sustainable growth; 2. instruction provided throughout all societal settings and at all levels (family, school, job, community); 3. education that helps people become responsible citizens and advances democracy by enabling people to exercise their rights and carry out their obligations; 4. instruction based on the idea of continuous learning; and 5. instruction that promotes a person’s holistic growth [16]. SDU works to develop students’ capacities to [17]:

- Possess a comprehensive and methodical way of thinking that allows them to understand the diversity of social challenges and occurrences;
- Comprehend the principles of sustainable development, such as equality diversity, inclusivity, and human dignity;
- Consider options and use critical thinking;
- Gather data and evaluate it;
- Effectively converse with people.

In society 5.0, economic growth, technical advancement, sustainability, and human beings are all at the heart of changes. The SDGs may be actively supported by Society 5.0 through fostering wealth, eradicating poverty, and safeguarding the environment [18]. Humanity is entering the fifth social revolution, which forms the foundation of this innovative civilization model. New Society 5.0 technologies have had a significant impact on education in recent years, leading to important structural changes. Face-to-face instruction in the classroom is no longer required or necessary. With the use of videoconferencing and virtual reality tools, modern Society 5.0 technologies have promoted digital education, enabling simultaneous instruction of several students without the need for physical classroom space [19, 20]. In society 5.0, economic growth, technical advancement, sustainability, and human beings are all at the heart of changes. Humanity is entering the fifth social revolution, which forms the foundation of this innovative civilization model. The goal of the Society 5.0 idea is to approach societal problems in a novel way. With complete integration of big data, the Internet of Things (IoT), artificial intelligence (AI), and people services to support digital and physical infrastructures for humans, many components will be integrated in this new age of technology and a super intelligent society. Establishing social foundations that allow anybody to produce value at any time and everywhere, in a secure atmosphere that complies with natural surroundings, and without any constraints like the ones that now exist is the aim of this. All residents are expected to participate actively in a Society 5.0 (super smart society), increasing the integration of digital technology into various processes [21, 22]. In addition to smart technology, Society 5.0 offers a number of options that may contribute to the accomplishment of the aforementioned objectives and lead to developments that strengthen sustainability [23]. Sustainable development goals (SDGs) from the standpoint of Society 5.0 allow for a new biosphere in which people live prosperous and fulfilling lives in accordance with social, economic, and technical advancement without endangering the environment [24]. Most nations should focus their investment strategies and research toward a similar model of development, where smart infrastructure contributes to sustainable development and, consequently, to the establishment of Society 5.0 in the future [23, 25], because the objectives and measures pursued by Society 5.0 are compatible with the Sustainable Development Goals (SDGs) [20]. In order to create wealth, eradicate poverty, and safeguard the environment, Society 5.0 may actively promote the SDGs [26]. Smart towns, smart cities, and smart universities are products of the technologically advanced civilization [19, 26].

## 3. Research methodology

The aim of the article is to identify the opportunities and challenges facing the surveyed universities in the context of their pursuit of sustainable development and building a 5.0 society based on opinions obtained from students of these universities from 10 countries around the world. The research sample in this study represents a subset of university students spanning 10 countries worldwide. These students were carefully selected to gather data and evaluate perceptions concerning various aspects related to the university’s progression towards sustainable development and the development of Society 5.0. Students should be considered as one of the main stakeholders in the university’s sustainability activities, actively collaborating with the university on this issue. These are people who actively participate in the life of the university, use its resources, and influence its atmosphere and culture. Their awareness, involvement, and opinions on sustainable development can significantly influence universities’ shaping of sustainable development strategies and initiatives. Therefore, the research took into account the perspective of students, and their opinions were treated as input into the decision-making process regarding sustainable development at the university. The research sample encompassed students from both private and public universities across diverse countries, enrolled in undergraduate, graduate, and doctoral programs, pursuing a wide array of academic disciplines. The research sample was international and diverse, reflecting a wide range of cultural and social perspectives. This sample was used in the research analysis to identify discrepancies in the effectiveness of raising student awareness of sustainability between public and private universities.

The survey was conducted among university students in 2022, from April to December. The research was conducted using a Computer-Assisted Web Interviewing (CAWI) questionnaire. This online survey facilitated researchers in efficiently reaching a diverse and geographically dispersed group of students. The CAWI questionnaire was distributed to students at a number of universities, and those who responded on a voluntary basis made up the research sample. This approach ensured the inclusion of a broader spectrum of students from different countries and universities, providing valuable information on universities’ road towards SD and Society 5.0.

The results outlined in the article are a segment of a comprehensive survey, that was designed to gather data and insights for comprehending, evaluating, and analysing the influence of the surveyed universities on sustainable development within the framework of constructing Society 5.0. The study was titled: "University 4.0 Sustainability towards Society 5.0". The survey questionnaire comprised 21 closed-ended questions and 8 questions for analysing the respondents. The survey questionnaires were distributed to students in 13 countries globally, including 9 from Europe and 4 from Asia. A total of 301 students provided their answers.

Questions were selected in detail from the survey (11 in total) to effectively illustrate the opportunities and challenges that the surveyed universities face on their path towards sustainable development and building Society 5.0. The survey questions selected for research analysis in this article are:

- Define what the University of Sustainable Development means to you?
- Thinking about your time in education so far, which of the following topics have you included in your teaching?
- How do you rate your current place of study in relation to its implementation of specific pro-environmental and sustainable development activities?
- Thinking about your time in education as a whole, which place where you studied most encouraged you to think and act on behalf of the environment and other people?
- Do you think that students choosing a university should check its sustainability activities?
- Are the actions taken by your university towards sustainable development important to you?
- Are you proud of the initiatives undertaken by your university for sustainable development?
- Do you think the university can do even more for sustainable development?
- Which pillars of sustainability do you think are most important for universities?
- Which pillars of sustainable development do you think are the most difficult for universities to implement?
- Do you think universities are effective in building students’ awareness of sustainable development?

The answers to these questions will provide information regarding students’ perspectives on the importance of sustainable development for universities, the assessment of current pro-ecological activities of universities, the role of information about sustainable development in choosing a university, the level of involvement and awareness of the student community in this issue, and the perception of the effectiveness of universities in educating about sustainable development. These responses will provide insight into both the opportunities and challenges facing universities in the context of SD and building Society 5.0.

A statistical analysis of the collected results was performed using a pie chart to identify the profile of a typical respondent ‒ a student participating in the research, and using a series of column charts to analyse the percentage structure of respondents’ answers to the analysed survey questions. A research hypothesis was also formulated that public universities are more effective at building students’ awareness of sustainable development than private universities. To verify this hypothesis, the non-parametric Pearson chi-square test dedicated to 2x2 tables and the PQStat program from PQStat Software were used. With the assumed significance level of α = 0.05, the hypothesis was tested.

Research limitations that should be pointed out are time constraints, which are related to the fact that the study was conducted in 2022 and the perspectives on sustainable development and society 5.0 may have changed since then. Another limitation is territoriality, as the study focused only on students from Europe and Asia.

The article can help the universities under study in identifying opportunities and challenges related to SD and on the way to building Society 5.0, and in developing effective strategies that integrate SD goals with the mission, educational programs and activities of the academic community. Ultimately, it can support university decision-making and policy & strategy-making, encouraging active involvement in global social and development initiatives.

The article fills a gap in research regarding the role of universities in the context of sustainable development and society’s transformation towards Society 5.0, thereby contributing to the existing literature. By analysing students’ opinions, universities’ approaches and the differences between public and private universities, the article points to specific areas where universities can take action and potential challenges that they must effectively overcome. By examining public and private universities, the study sheds light on the effectiveness of these institutions in raising student awareness, providing universities with valuable comparative insights to improve their sustainability efforts. Thanks to this, the article makes a concrete contribution to understanding how universities can actively support the development of a sustainable society and adapt to the concept of Society 5.0. This information can help universities implement effective sustainability initiatives.

## 4. Results and discussion

**Fig 1:**
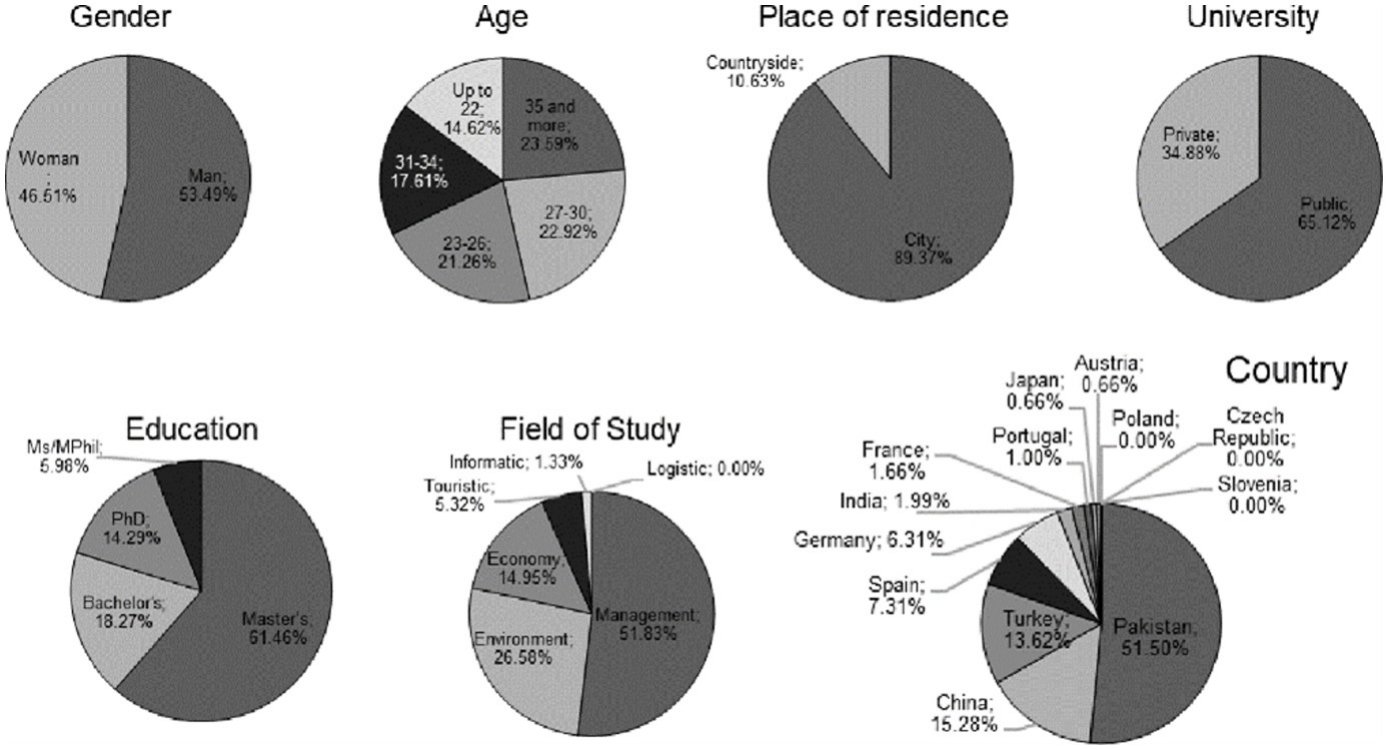
Percentage structure of respondent characteristics—University students.

The results of the analysis of the percentage structure of the characteristics of respondents ‒ students studying at the surveyed universities are presented in Fig 1.

Based on the percentage structure of respondent characteristics, it can be identified the profile of a typical student studying at the analysed universities. The typical student is a male, aged 35 or older, residing in an urban area, seeking education at a public university for a second-degree level (master’s degree), with a major in management, and hailing from Pakistan. Analysis of the typical student profile suggests that SD universities must effectively differentiate their educational approaches to meet the expectations and needs of different student age groups, including those aged 35 and older. The universities analysed should adapt their educational programs and teaching approaches to the profile of typical students studying at their institution in order to better respond to their requirements in the context of SD activities. There may be a difference in the perception of the importance of sustainable development activities and initiatives among students of different ages and from different social and demographic groups, which the surveyed universities should note.

**Fig 2:**
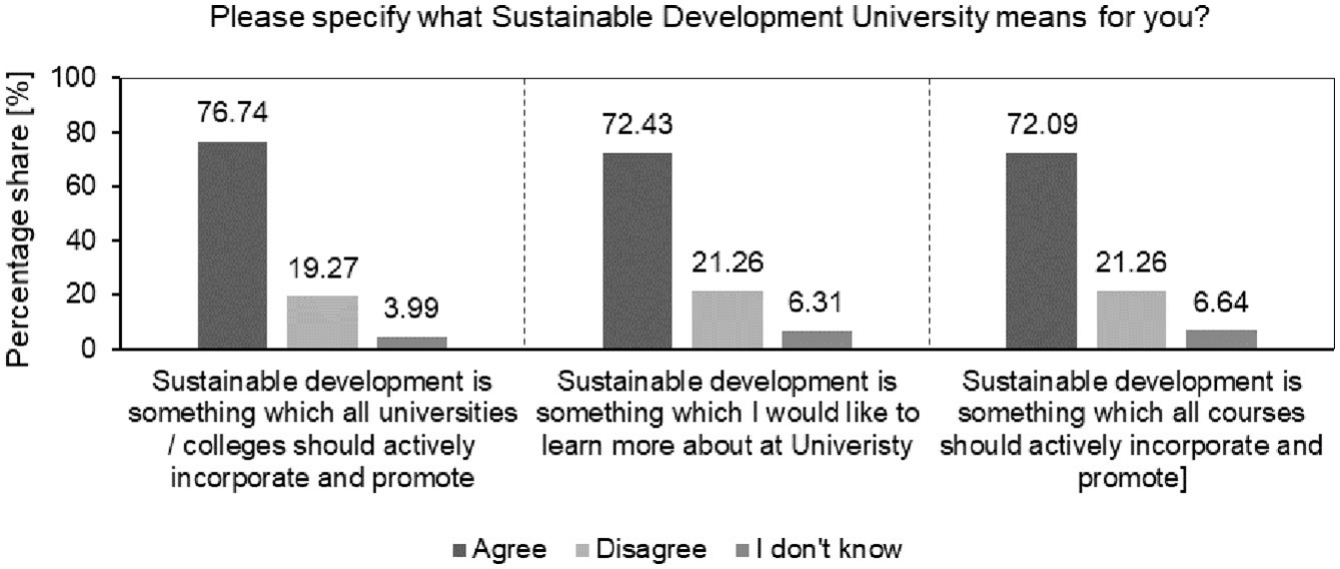
Analysis of the percentage structure of students’ answers to the question about what the SDU means to students.

The result of the analysis of the percentage structure of answers to the question about what the SDU means to students is presented in Fig 2.

The result clearly indicates that students want SD to be actively included in curricula and promoted by their universities (76.74%), they want to learn more about this concept in various courses, through various initiatives implemented by their universities (72.43%) and they also want all courses at their universities to actively take into account and promote this concept (72.09%). The results indicate significant student interest and demand for sustainability to be comprehensively integrated into their university experience. This provides a clear opportunity for the analysed universities to align its initiatives and educational offerings with the principles and goals of SD. By leveraging these opportunities, the analysed universities should position themselves as a catalyst for social transformation, educating students who are not only literate in sustainability topics but also equipped with the knowledge, skills, and mindset to contribute meaningfully to the Society 5.0 vision of a human-cantered, sustainable and technologically advanced society.

**Fig 3:**
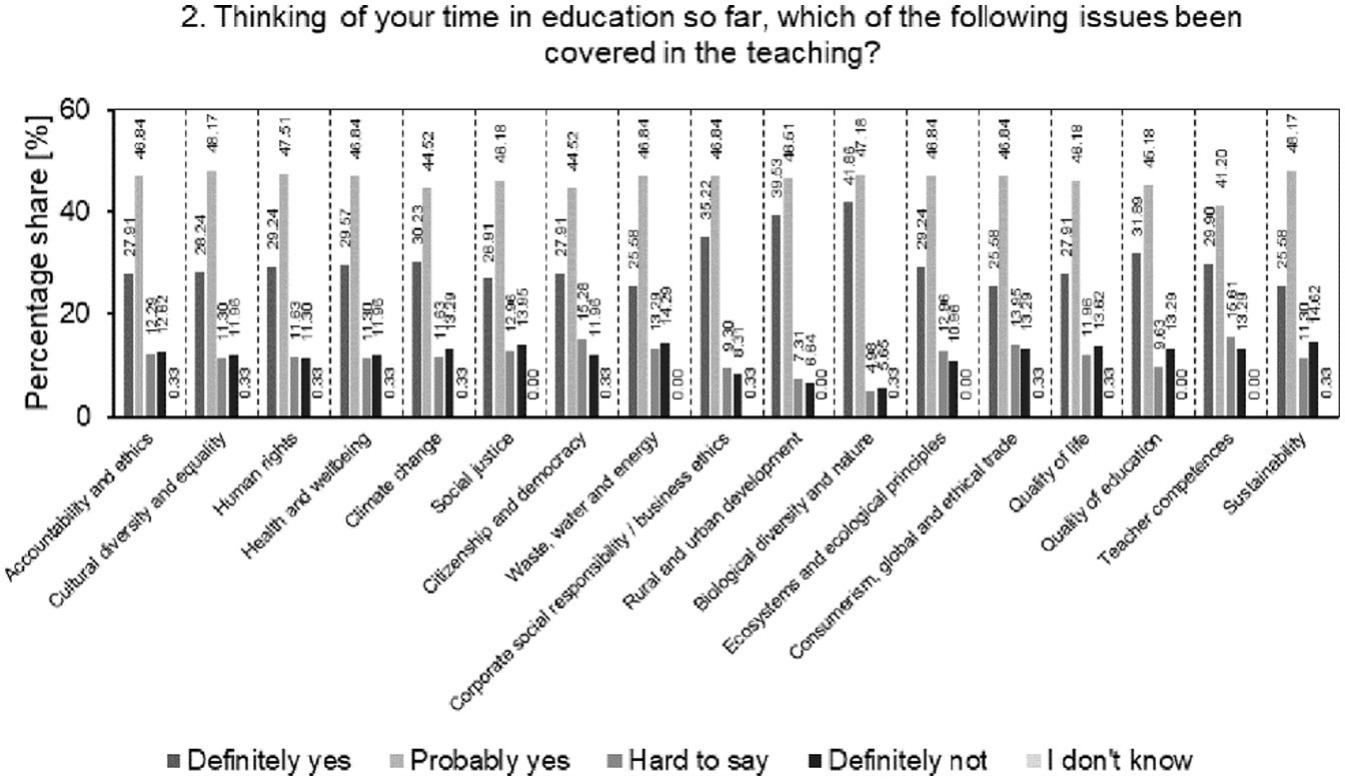
Analysis of the percentage structure of students’ answers to the question of which issues important for SD have been included in the teaching process so far.

The result of the analysis of the percentage structure of students’ answers to the question of which of the SD issues (important for the development of Society 5.0) have been included in the teaching process so far is presented in Fig 3.

Students’ strong belief in the presence of all these issues in the curriculum is 30.15%, while students are more likely to believe that these issues were discussed in the curriculum (average level: 46.26%). The answers "difficult to say" and "definitely not" account for an average of 23.39% of the answers. The average level of students recognizing that various sustainability issues had previously been covered in the curriculum suggests that there was some engagement at different levels of education in these aspects. This represents an opportunity for universities to increase the integration of these topics in their curricula and meet the growing expectations of students in this topic. The share of "difficult to say" and "definitely not" answers (on average 1/4 of all answers) may indicate the need for greater clarity and intensification of aspects of SD in education. The results from this question should be interpreted as an opportunity for the surveyed universities to create a system of better communication and education on various sustainable development issues in order to eliminate students’ doubts and ambiguities regarding the presence of these issues in their curricula. By adapting their curricula, promoting innovation and interdisciplinary collaboration, and strengthening communication with students to better understand and meet their sustainability needs, the universities studied can respond to these growing expectations.

**Fig 4:**
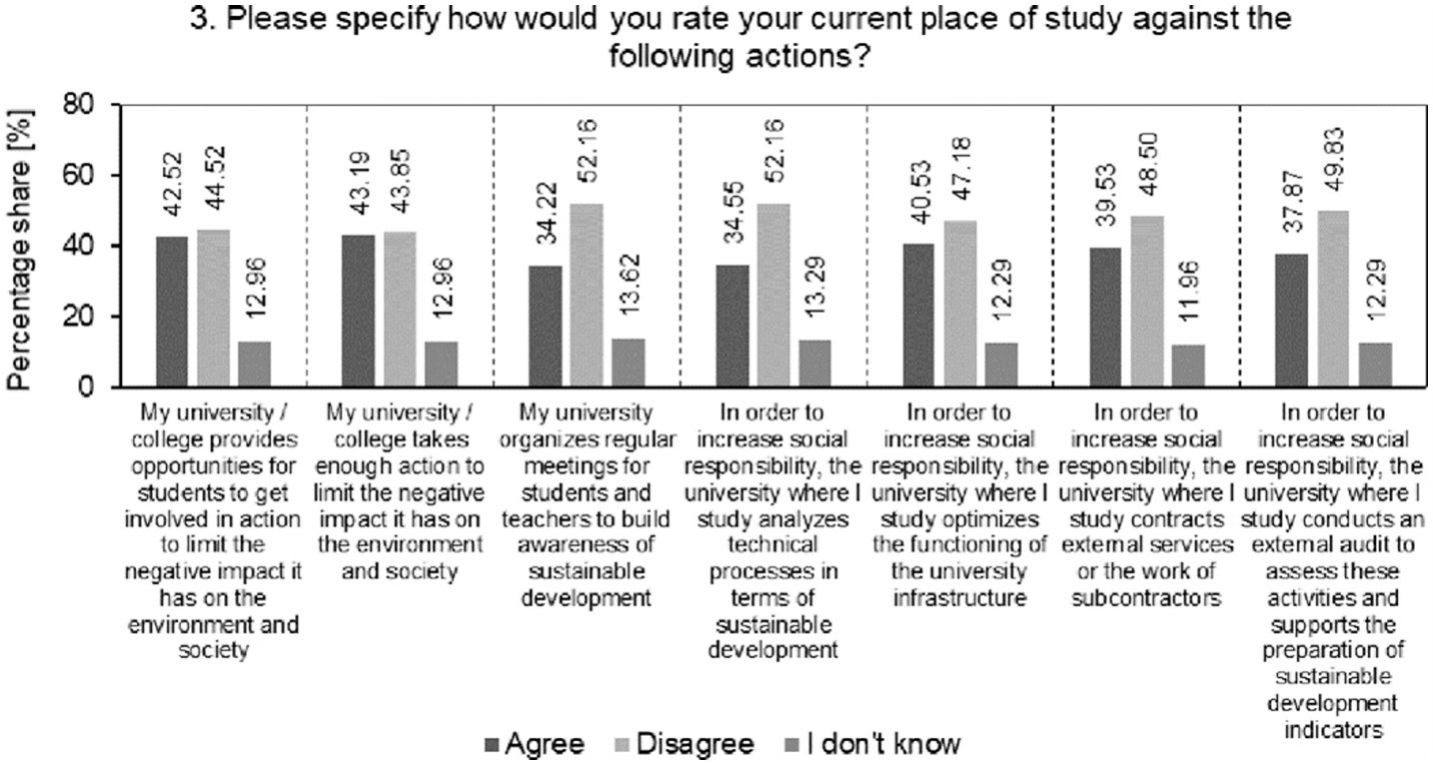
Analysis of the percentage structure of students’ answers to the question how they evaluate their current place of study in relation to specific actions.

The result of the analysis of the percentage structure of students’ answers to the question how they evaluate their current place of study in relation to specific actions is presented in Fig 4.

The analysis shows that the overwhelming percentage of students do not agree with the statements regarding possible actions taken by their universities in the area of SD. This may suggest either a lack of actual activities in this area or an inappropriate way of communicating these activities to the target group ‒ students. The highest level of "disagreement" among students concerns the statements that the university organizes regular meetings for students and lecturers to build awareness of sustainable development and that the university where they study analyses technical processes in terms of sustainability (52.16% each). The universities surveyed should analyse the root causes of such student responses. They should also focus on improving communication with students about their sustainability activities, as there is always room for improvement in this area. This may include clearly presenting your initiatives, activities and achievements in this area through the use of various visual management tools (Knop, 2020) such as large screens, televisions, bulletin boards, website announcements, posters and others. The surveyed universities should organize regular meetings, workshops and even social campaigns aimed at sustainable development, which will encourage students to be more involved and build awareness of social responsibility. It should be valuable for the universities surveyed to analyse and evaluate their technical processes from a sustainability perspective. This will enable the implementation of technological innovations consistent with the concept of Society 5.0. Encouraging cooperation between various university departments by their management, between different fields of study and students of these fields will allow for a holistic view of the effectiveness of sustainable development activities at the university. Interdisciplinarity and synergy of "good practices" at various faculties will allow us to intensify activities in the field of SD at the surveyed universities.

**Fig 5:**
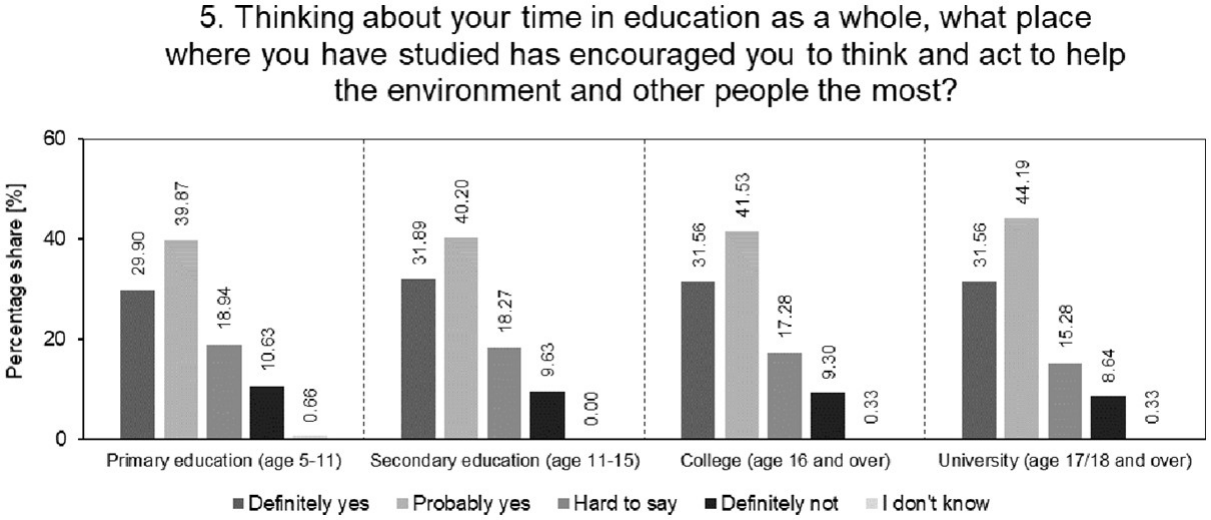
Analysis of the percentage structure of students’ answers to the question of how they evaluate different levels of education in encouraging thinking and acting for the benefit of the environment and other people.

To assess the extent to which universities inspire students, as opposed to lower levels of education, to think and act for the benefit of the environment and society, one question was analysed. The results of this analysis are presented in Fig 5.

As can be seen in Fig 5, universities from all educational paths, in the opinion of students, best promote thinking and acting for the benefit of the environment and other people (a total of 75.75% of answers of the type "definitely yes" and "probably yes"). However, the confidence in this is not high (only 31.56%). This may be due to the fact that students may not have full knowledge of the activities undertaken by their universities in the field of sustainable development. They may be aware of initiatives but lack visibility into the full scope of activities and their effectiveness. The universities studied therefore have the opportunity to increase transparency and communication regarding their sustainability initiatives, which may increase the level of trust and awareness among students. It is also necessary to increase the commitment and effectiveness of sustainable activities by universities to build students’ confidence in the positive impact of these activities on the environment and society.

**Fig 6:**
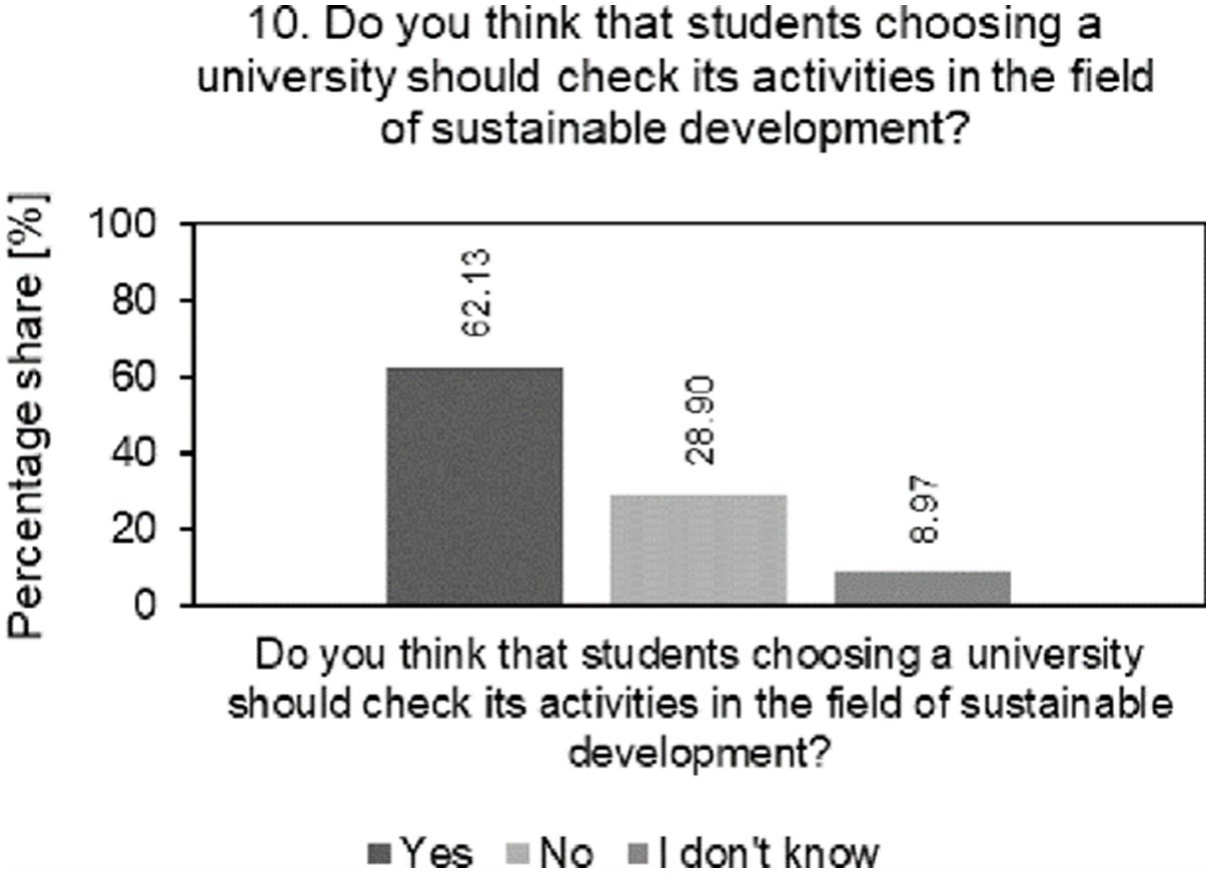
Analysis of the percentage of students’ answers to questions regarding the importance of SD activities in choosing a university.

Results of the analysis of the percentage structure of students’ answers to the questions whether students, when choosing a university, should check its activities in the area of SD is shown in Fig 6.

As shown in Fig 6, 62.13% of students indicate that they consider university activities in the field of SD as one of the factors when choosing a given university. This is the result of growing social interest and ecological awareness of students around the world, which, among others, prompts them to look for universities that care about sustainable development. Students are becoming more and more aware of SD, paying more and more attention to the social responsibility of universities and their approach to environmental issues when choosing a given university. By choosing a university that is actively committed to sustainability, students can feel that their choice has a positive impact on society and the environment. Therefore, it is very important that sustainability becomes an important part of the strategies of the universities surveyed and that universities more actively promote their environmental initiatives, social responsibility and sustainable practices. Such activities may attract students who, according to research, want to participate in this type of initiatives and be part of a university committed to having a positive impact on the world.

**Fig 7:**
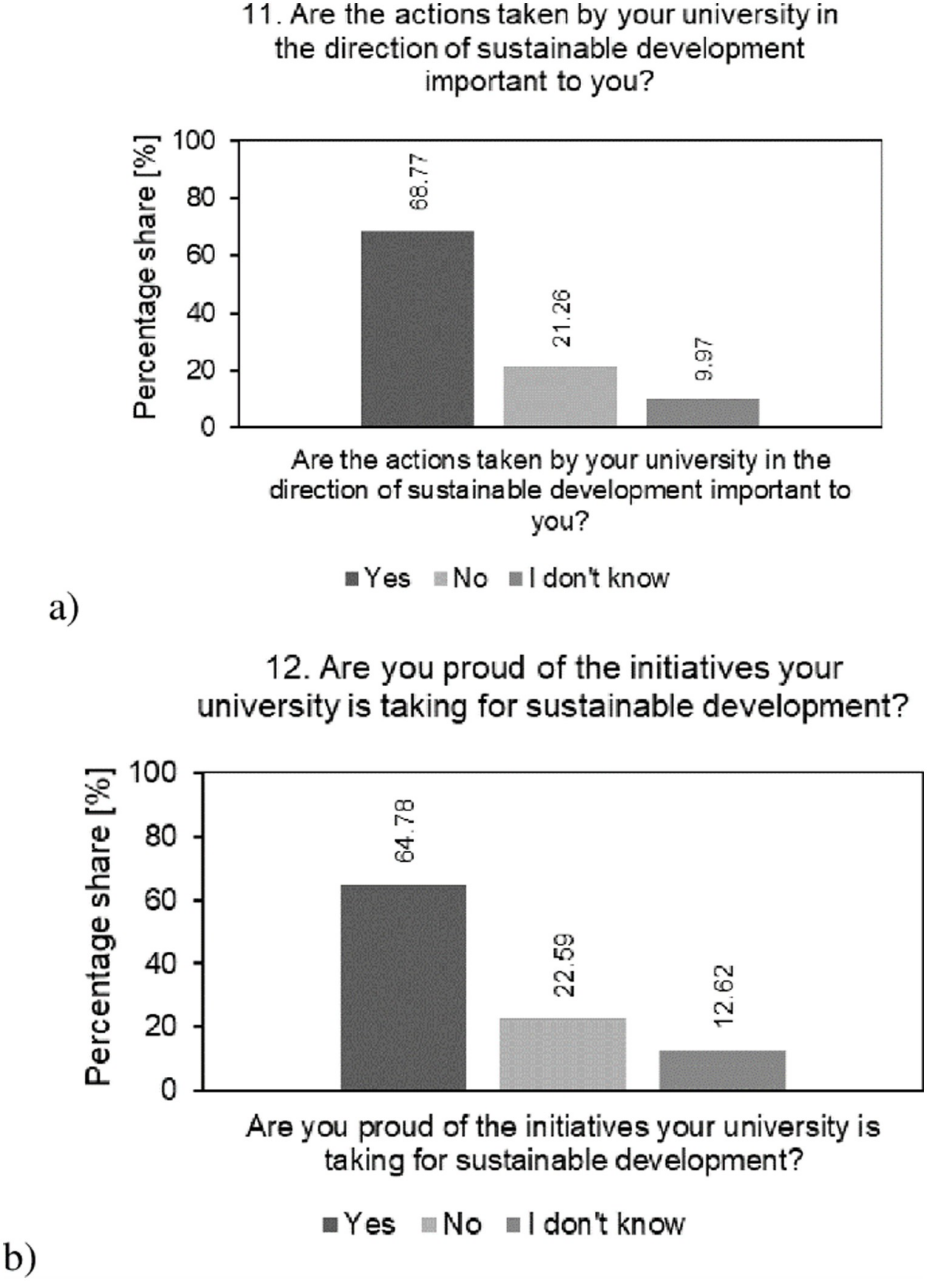
Analysis of the percentage of students’ responses to questions regarding: a—their perception of the importance of activities; b—satisfaction with SD initiatives undertaken by the university during their studies.

Fig 7 shows the result of the analysis of the percentage structure of students’ answers regarding the perception of the importance of activities undertaken by universities in the area of SD (Fig 7a) and their own satisfaction with the initiatives implemented by universities in this area (Fig 7b).

The results of the analysis indicate that a significant percentage of students consider university activities for SD to be important (68.77%) and express pride in the initiatives undertaken by universities in this area (64.78%). Thus, a proactive approach to sustainability can contribute to the attractiveness of universities in the eyes of potential students. Positive student reactions should motivate the researched universities to further develop and expand SD initiatives. At the same time, maintaining a high level of activity and effectiveness of SD activities may be a challenge for the surveyed universities to meet students’ expectations.

**Fig 8:**
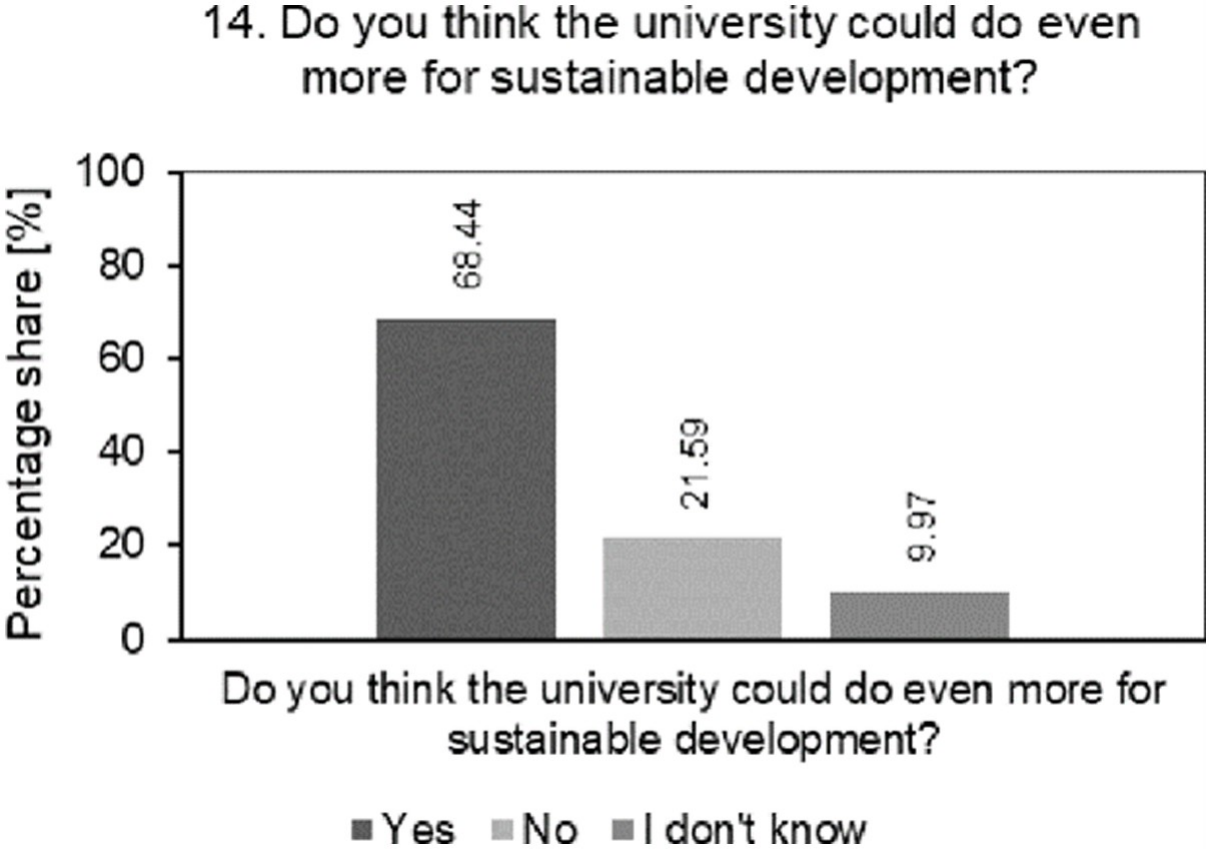
Analysis of the percentage of students’ answers to question about whether they believe that the university they study at can do even more for SD.

Fig 8 shows the result of the percentage analysis of structure of students’ answers to the question whether they think that the university where they study can do even more for SD.

68.44% of students believe that universities can make greater efforts towards SD. Students, increasingly aware of global challenges related to SD, expect greater involvement of their universities in these issues. Education and the growing social awareness of students about the impact of individuals and institutions on the environment and society make them expect greater involvement of universities in sustainable development. The universities surveyed therefore need to invest in education, training and information campaigns to raise students’ awareness in this area. It is necessary to take SD into account as an integral part of the strategy, mission and development plans for the universities under study. This must be visible and noticeable in all aspects of the university’s functioning. The universities surveyed must also strive to create a culture that promotes sustainable development and a socially responsible approach. It is also important to obtain stable and long-term financing for SD projects. The surveyed universities should also invest in research and development of sustainable technologies and promote innovations that contribute to the protection of the environment and society. Such activities and their effective communication should be noticed by students and improve the perception of the surveyed universities as an institution promoting SD.

**Fig 9:**
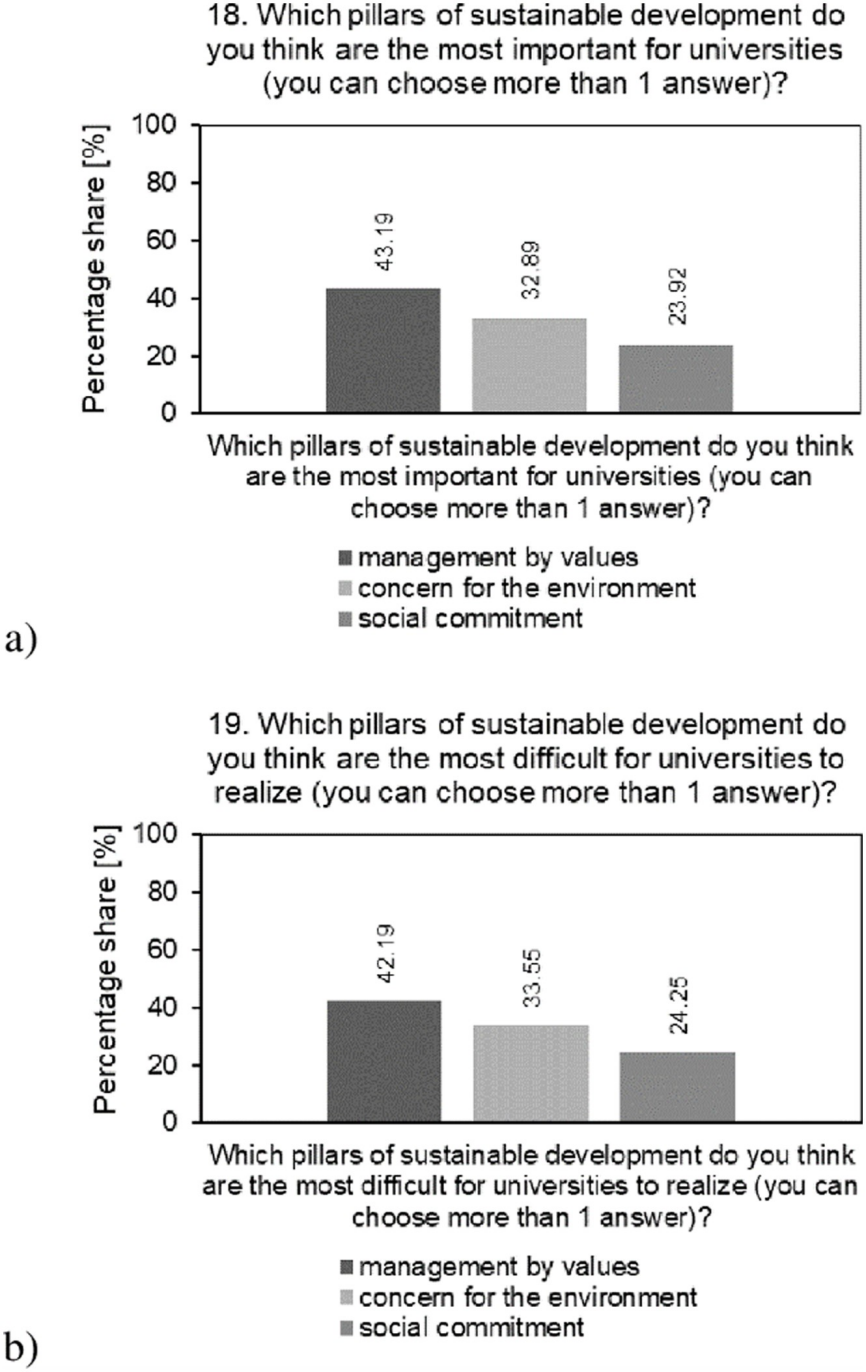
Analysis of the percentage of students’ answers to questions regarding: a—their perception of the importance of the SD pillars for SDU; b—the difficulties of their implementation by SDU.

The results of the analysis of the percentage structure of students’ answers to questions regarding their perception of the importance of the SD pillars for their university (a) and the difficulties in their implementation by their university (b) are presented in Fig 9.

The analysis shows that the most important pillar of SD according to students for the surveyed universities is management by value (43.19%). At the same time, it is the pillar that students consider the most difficult for universities to implement. Second in the hierarchy of pillars was concern for the natural environment (32.89%), and third was social involvement (23.92%). It is worth noting that these two pillars also seem difficult for students to implement, ranking second (32.89%) and third (24.25%), respectively. The surveyed universities should focus their efforts on improving and promoting the concept of value-based management. They should strive to balance diverse interests to create lasting and sustainable value for all their stakeholders. The surveyed universities must also take action to identify obstacles and develop strategies for the effective implementation of the value-based management concept. They should establish long-term development strategies that take into account both short-term economic goals and long-term social and environmental goals. The organization by the surveyed universities of social campaigns, educational events, volunteering and other activities promoting sustainable development and informing stakeholders about initiatives, goals and progress in the field of sustainable development is the key to building trust and engaging the student community. The implementation by the surveyed universities of technological innovations aimed at improving durability, energy efficiency and environmental protection will also be key in this area.

**Fig 10:**
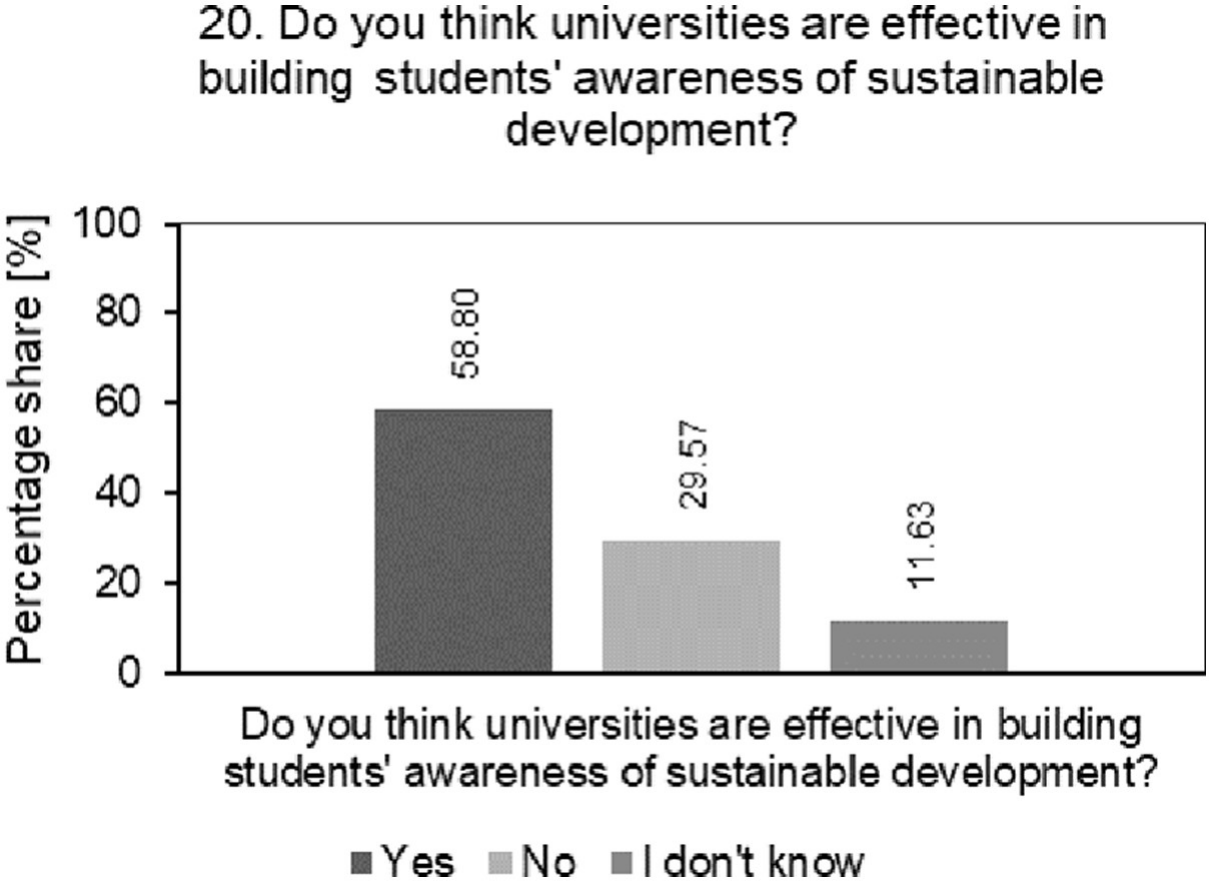
Analysis of the percentage of students’ answers to questions about whether universities are effective in building students’ awareness about SD.

Fig 10 shows the result of the analysis of the percentage structure of students’ answers to questions about whether universities are effective in building students’ awareness of SD.

Almost 60% of surveyed students believe that their universities effectively build awareness of sustainable development. This result indicates that universities already have some influence on the awareness of SD among students. Although the results are positive, they suggest that the surveyed universities still have a lot to do in the area of sustainability education. They should intensify educational activities, including sustainability in more curricula and organizing educational events and workshops. Although universities can be effective in educating students in the area of SD, they may face challenges in effectively communicating these activities. It is worth analysing current communication channels, assessing their effectiveness and taking improvement actions in this area. The efforts of the surveyed universities in the area of sustainable development should be more visible and understandable to the academic community and wider society. The challenge of maintaining the pace of implementation of activities in the area of SD and continuous improvement of these activities so that they have a real impact on the awareness and involvement of the academic community is a significant challenge for the surveyed universities.

It was verified whether the level of effectiveness in building SD awareness among students depends on the type of university in terms of legal status and form of ownership (public vs. private university). The following hypotheses were verified:

- H_0_: there is no relationship between the legal status and form of ownership of the university and the level of students’ awareness of sustainable development;
- H_1_: there is a relationship between the legal status and form of ownership of the university and the level of students’ awareness of sustainable development.

**Fig 11:**
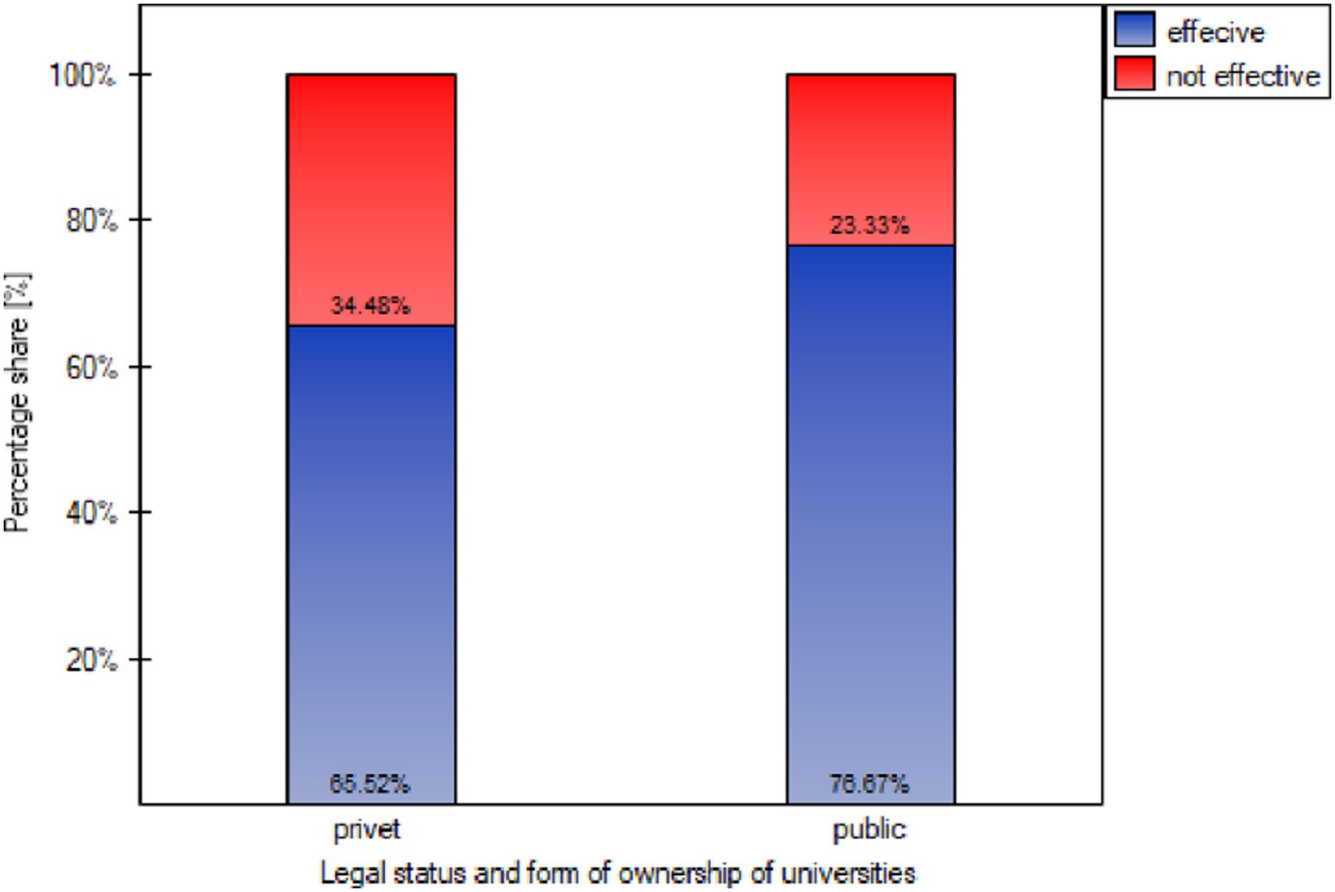
Stacked column chart in rows of the percentage of students’ responses in terms of assessing the university’s effectiveness (yes ‒ 1/effective, no ‒ 0/ineffective) in building students’ awareness of sustainable development depending on the type of university (private vs. public).

The hypotheses were verified using the nonparametric Pearson Chi-square test for 2×2 tables [27]. The table of expected frequencies did not contain values less than 5, so the Cochran condition was met, which allowed the use of this test. At the adopted significance level of α = 0.05, the Pearson Chi-square test confirmed the truth of the null hypothesis (p = 0.266628). There is no relationship between the status and form of ownership of universities and their effectiveness in building students’ awareness of SD. It should therefore be concluded that the status and form of ownership of a university do not significantly determine the effectiveness of the university in building students’ awareness of SD, which is confirmed by the results of the percentages of students’ answers presented in a stacked column chart by row (Fig 11).

The lack of a significant relationship between the status and form of ownership of universities and their effectiveness in building awareness of sustainable development among students indicates that the organizational and legal conditions in which private and public universities operate have no influence on the perception of their activities in the field of SD among students. It is also, in a sense, an encouragement for mutual cooperation between various universities, analysis of local needs and continuous improvement. The surveyed universities, regardless of their legal status and form of ownership, can share experiences and strategies that have proven effective in building awareness of sustainable development. Creating a platform for collaboration between different types of universities can lead to mutual enrichment and a better understanding of what works best. The surveyed universities, regardless of their status, should adapt their activities in the field of building awareness of sustainable development to local needs and socio-cultural conditions. They should also constantly improve their approach to building awareness of sustainable development by periodically assessing SD activities and taking effective first-time corrective and improvement actions (pre-emptive actions). Innovation and adapting university strategies to the needs of various stakeholders (including students) is the key to maintaining and increasing effectiveness in building environmental awareness of SD.

## 5. Conclusions

The aim of the article was to determine what opportunities and challenges universities face in the context of striving for sustainable development and building Society 5.0 based on the results of surveys conducted at universities from 10 countries among their main stakeholders ‒ students. In the course of the research and analysis of results, the following conclusions can be drawn in this regard:

- The results indicate the growing interest of students in the issues of sustainable development, which is a potential opportunity for universities to integrate and intensify activities in this area;
- The concept of value management in the context of building sustainable development at universities is crucial in the opinion of students, but it is also an implementation challenge;
- Education, implementation of pro-environmental activities, effective communication about these activities at universities are the key to increasing the awareness of the academic community about sustainable development;
- Introducing sustainable development at universities requires adapting strategies, interdisciplinarity and mutual cooperation of universities across divisions, long-term financing and effective evaluation of activities.

Summarizing the research results and analysing the opportunities and challenges for the researched universities in the context of SD and striving to build a Society 5.0, the following key conclusions can be distinguished:

Chances:

- Increased interest in sustainability: students show increased interest in sustainability issues. This opens up the possibility of attracting more students by actively engaging universities in these issues;
- Effective communication, pro-environmental activities, education: effective communication of sustainable initiatives and education in this area among students can bring positive results and increase the level of involvement of the academic community. For strategic communication, universities can use visual management tools;
- Introducing technological innovations: technological innovations in the context of sustainable development can become an important pillar of a university’s strategy, encouraging high school graduates to choose a university and building a positive image of the university among students and other stakeholders.

Challenges:

- The need to improve educational activities: the challenge is to improve educational activities so that sustainability becomes an integral part of curricula and students’ awareness;
- Adaptation to local realities: universities must adapt their sustainability initiatives to local realities so that they are effective and relevant to the specific needs of the community;
- Value management and long-term financing: introducing the concept of value management in the context of sustainable development requires strategic management and long-term financing of projects;
- Work beyond divisions: cooperation between university departments, between different universities, regardless of the status of the organization and the form of ownership in the field of exchange of experiences, application of best practices will ensure a synergy effect and will be a catalyst for changes;
- Enabling interdisciplinarity and engaging the academic community: the challenge is to incorporate an interdisciplinary approach and involve the entire academic community in sustainable development initiatives;
- Routinised improvement of the effectiveness of sustainable initiatives: there is a need to continuously monitor and evaluate the effectiveness of sustainable initiatives to ensure that they are effective and achieve their intended goals;
- Continuous improvement: maintaining the pace of change in the area of implemented initiatives and implementation of sustainable development goals and introducing newer, more effective practices in the field of sustainable development will be an indicator of the continuous development of the universities and will help accelerate the process of building a conscious Society 5.0.

To sum up, the studied universities have a chance to attract attention and engage students by actively promoting sustainable development. However, this requires a balanced approach, improved educational activities, and financial support. Effective value management, technological innovation, active participation, and action beyond the divisions of the academic community are the keys to achieving these goals. Additionally, fostering a culture of sustainability within the university ecosystem and transparently communicating these initiatives to students and the wider community will allow universities to be noticed, appreciated, and motivated, which can further increase university engagement and participation in sustainability efforts.

A key component of the World Bank’s 2030 sustainable development plan is Goal 4 (SDG4), which calls for high-quality education. According to Za-hid et al. [28], many universities in underdeveloped nations still do not implement and adopt sustainable practices. In underdeveloped nations, SDU faces a variety of challenges, such as inadequate institutional support and governance [29]. The failure of sustainability in the SDU of poor nations may be attributed to a number of reasons, including a lack of commitment, preparedness, consensus, guidelines, planning, leadership, infrastructure, resources, innovation, technology, and training and development [28]. But according to Ad-Ams et al. (2018), SDUs in underdeveloped nations still haven’t included sustainability into their operations, planning, culture, or curriculum. Students might also be made more conscious of sustainability via university education [30]. Through the beginning and development of their teaching, curriculum, research, campus operations, community outreach, and daily activities, SDU integrates sustainability [31]. Quist and Tukker [32] contend that government, educational, and financial actors must work together in order to achieve sustainability, which in turn depends on a substantial interconnection of innovation, learning, and collaboration. In order to address the challenges of the shift to sustainability, Van de Kerkhof and Wieczorek [33] recommend a multi stakeholder learning and cooperation strategy. The five primary components of sustainable development ‒ governmental, economic, social, environmental, and technological ‒ are where Society 5.0 innovations might improve it. It is conceivable that for sustainable development, technology advancements brought forth by Society 5.0 have changed how people live, work, and even operate in organizations. Technology advancements are used in these human-centered innovations to improve human wellbeing. Smart transportation, automated driving systems, smart healthcare, and even internet shopping have all contributed to an improvement in human well-being [34].

Planned future research on the role of universities in striving for sustainable development and building Society 5.0 will be based on comparative research between surveyed universities from different countries in order to identify global trends, best practices and cultural differences affecting the effectiveness of activities and the implementation of setting goals in the area of sustainable development. development. Models of sustainability education programs that are particularly effective in a given country will be identified, which can help adapt and improve national education programs. Research in these areas can provide valuable tips and information for universities in a given country, which will allow for more conscious and effective planning and implementation of sustainable development strategies, consistent with global trends and best national practices.
